# A Qualitative Study on the Experiences of Preclinical Students in Learning Clinical and Communication Skills at a Simulation Centre

**DOI:** 10.1007/s40670-023-01851-7

**Published:** 2023-09-07

**Authors:** Chong Pek Sam, Joann Lalita Nathan, Jacintha Anita Aroksamy, Nithia Ramasamy, Norul Hidayah Binti Mamat, Vishna Devi Nadarajah

**Affiliations:** 1 Clinical Skills & Simulation Centre, International Medical University (IMU), Kuala Lumpur, Malaysia; 2 IMU Centre for Education, International Medical University (IMU), Kuala Lumpur, Malaysia; 3 Department of Human Biology and IMU Centre for Education, International Medical University (IMU), Kuala Lumpur, Malaysia

**Keywords:** Simulation centres, Learning resources, Preclinical medical students’ experiences, Kolb’s experiential learning cycle

## Abstract

**Introduction:**

Simulation centres (SC) and its learning resources are now firmly established as part of medical education. In SC, medical students obtain both knowledge and skills based on a combination of theory and practice using provided resources. This study aims to explore medical students’ use of SC learning resources to learn clinical and communication skills based on Kolb’s experiential learning cycle. This is based on the research question ‘How are the SC resources useful in supporting preclinical medical students’ clinical and communication skills learning?’ The findings of the study can make a case for further enhancement of SC design and resources for medical students in the preclinical phase.

**Methods:**

A qualitative study involving 20 preclinical medical students with learning experiences in SC was conducted between December 2019 and 2020 at a medical school in Malaysia. Semi-structured interview questions were developed based on Kolb’s learning cycle. The data were thematically analysed using the six phases of Braun and Clarke’s thematic analysis.

**Results:**

Three main themes were identified based on preclinical medical students’ experiences in SC; they were ‘preparation for authentic clinical experience’, ‘accessibility of multiple resources for learning and support’ and ‘opportunities to learn and improve’.

**Conclusions:**

The SC’s resources have a significant and positive role in supporting preclinical medical students learn clinical and communication skills. The SC resources prepared them for authentic clinical experiences with a patient-centred care approach and self-directed learning opportunities. Social support from peers, peer tutors and academics emerged as a key finding and resource of the SC as they help preclinical students learn and improve.

## Introduction

Simulation centres (SC) and their learning resources are now firmly established as part of medical education and clinical teaching. These learning resources include learning spaces such as examination rooms, simulation labs, control rooms and briefing/debriefing rooms. There are also resources in the form of learning materials and aids like mannequins, task trainers, CCTV recordings, augmented reality (AR) or virtual reality (VR) software and gadgets in SC [1]. Learning resources also take the form of a group of experts, including simulated patients (SP), faculty [2] and peer tutors [3] who aid medical students in learning clinical skills that encompass history taking, physical examination, use of diagnostic reasoning, effective communication and teamwork. These resources are utilised collaboratively to coordinate and deliver services in SC [2].

Compared to classroom education, training in SC occurs in carefully designed clinical learning environments that are influenced by the curricular needs and bring about multiple advantages to student learning outcomes [4, 5]. The training consists of a structured teaching concept under guided supervision in consideration of methodological psychosocial medicine and educational ideas, ideally creating an atmosphere that allows repeated, anxiety and risk-free practice of targeted skills [6]. In SC, medical students obtain knowledge based on the combination of theory and practice using provided resources. These resources which include the previously mentioned learning spaces that are well-equipped simulation materials and learning aids allow students to recall, apply and incorporate relevant theoretical knowledge in managing patients [7]. Other advantages of SC resources include a thorough preparation of medical students to practice clinical procedures in a safe setting before clinical clerkships, increasing self-confidence and establishing long-term effect on students’ competencies including communication and psychomotor skills [8, 9]. Furthermore, SC resources like SPs and peer assisted learning programmes can provide personalised assessment and constructive feedback to improve students’ competencies [2]. As more medical education activities are being carried out in SC, equipping SC with resources that meet contextual needs is vital for every institution [5].

Despite the advantages offered by learning at SC, some challenges can arise from faculty and students’ perspectives. For example, there are faculty concerns that time spent in SC takes time away from learning in authentic settings in healthcare and the community. Faculty are also concerned about the possibility of students using shortcuts such as omitting consent, safety procedures and not genuinely communicating with SPs [10]. Additionally, large student numbers translate into higher investment for high-fidelity simulation, staff numbers and space availability [11]. Students’ concerns with learning at SC include insufficient resources and facilities, lack of trained staff and accessibility of simulators [9]. The scenarios used during simulation and the role of simulated patient have been highlighted as challenges by students as being unreal or too extreme [12]. Nevertheless, literature shows that clinical skills training needs to be introduced gradually from year 1 of medical training to deliver an integrated curriculum. This means that students are introduced and trained in SC from year 1, which are the preclinical years [13].

A limited number of studies are currently found on preclinical students’ (usually years 1 to 3) experiences and benefits of using learning resources in SC as studies are more focused on the effectiveness of simulation-based learning in clinical and communication skills training [14–16]. This study aims to explore preclinical medical students’ use of SC learning resources to learn clinical and communication skills based on Kolb’s experiential learning cycle. This is based on the research question ‘How are the SC resources useful in supporting the medical students’ clinical and communication skills learning?’ The Kolb’s experiential learning cycle is used as the study framework because it describes a person’s learning experiences and progression via a cycle of four stages [17–19], concrete experiences (skills practised with the provided learning resources), reflective observation (reflection through the experiences from using multiple resources), abstract conceptualisation (initiate new concepts or modify what they have learned from the previous experience) and active experimentation (repetitive practice by using the learning resources to reach competencies). By exploring preclinical students’ experiences in the SC with the lens offered by Kolb’s, educators should be able to determine how the SC learning resources support effective learning of clinical and communication skills. The findings can help educators prioritise resources based on the learning needs and outcomes of preclinical students.

## Methodology

### Participants and Study Context

A total of 20 undergraduate medical students in their preclinical years consented to participate in this study. They have completed at least a year of study at IMU and have been using learning resources in SC for their learning in more than 50 sessions. The focus groups were conducted after they have completed the sessions. They were recruited through purposive sampling with the inclusion criteria of being in semesters 3 to 5 and enrolled full-time in the medical program. The distribution of the participants is demonstrated in Table 1.

**Table 1:** Distribution of participants

**Year**	**Semester**	**Number of participants**
**2**	**3**	**8**
**2**	**4**	**7**
**3**	**5**	**5**
**Total**		**20**

Semester 1 and semester, which are year 1 medical students, were not included as they have lesser exposure and experience to the learning resources in the SC compared to other preclinical students in semesters 3, 4 and 5.

The study was conducted at the International Medical University (IMU) in Kuala Lumpur. The medical program at IMU is a 5-year integrated and outcome-based medical program delivered in two phases: preclinical and clinical phases. The preclinical phase encompasses the first 2.5 years of the medical program and is delivered across five semesters. The preclinical phase curriculum includes learning about basic medical sciences, body systems, clinical skills including communication, history taking, physical examination and procedural skills.

The IMU SC provides learning resources such as a venue with closed rooms and ward settings, simulated patients, high-fidelity simulators and self-directed learning (SDL) space for students to learn clinical and communication skills. According to the timetable, a cohort of students attends small group teachings, roughly 1 or 2 teaching sessions per week for 14 weeks. Each session lasts for 2 h. A facilitator guides the student’s learning, and immediate feedback is given to improve their performance. Students practice clinical and procedural skills in the SDL space equipped with mannequins and educational videos at their own time after the teaching session. In addition, students commonly practice in pairs or group that encourages peer learning with feedback and comments.

### Data Collection

**Fig. 1:**
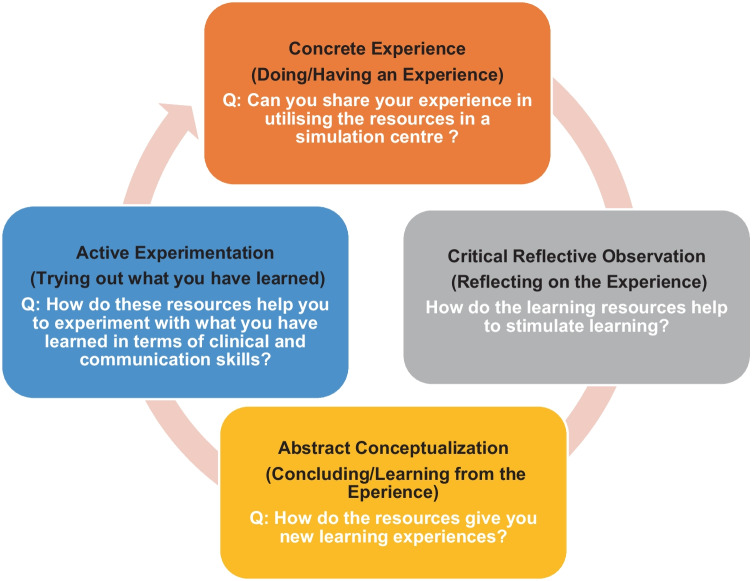
Semi-structure questions guided by the Kolb’s experiential learning theory

Recruitment and the data collection process started after approval by the IMU Joint-Committee on Research and Ethics (Approval No. 459/2019). Data was collected between December 2019 and 2020 through focus groups. There were students from semester 3 to semester 5 in each focus group, which is year 2 and start of year 3 students. Semi-structured interview questions were developed based on Kolb’s experiential learning cycle to explore participants’ recollections of experiences using the resources in an SC, detailed in Fig. 1.

Each focus group lasted between an hour to an hour and a half. It was carried out in three separate groups of 6 to 8 participants in a closed room and via the online platform of Microsoft Teams.

The focus groups were audio-recorded and guided by a protocol until data saturation was reached [20]. The recorded focus groups were then transcribed in a verbatim manner and analysed.

### Data Analysis

The data analysis was guided by Braun and Clarke six-phase thematic analysis [21]. The researchers repeatedly read the transcripts before generating initial codes. Then initial codes, ranging from *preparing for CSSC*, *availability of resources*, *improve skills*, *build confidence*, *hands-on practice*, *patient-centred care* and *realism and real-time feedback on performance*, were identified. The preliminary themes were then constructed by integrating components or fragments of ideas or experiences from the coded data, such as *preparation for authentic clinical experience*, *accessibility of multiple resources for learning and support* and *opportunities to learn and improve*. The preliminary themes identified through data coding that represented the context within the entire data set were reviewed and refined [21]. After the themes were formed, the researchers checked the association between the codes and the full data relevant to students’ experiences in utilising learning resources at SC to ensure consistency and cohesiveness of generated themes. When necessary, some themes were merged into one; some were split into different themes, some were discarded, and new themes were formed again [21]. Next, the researchers conducted and wrote a detailed analysis identifying the experiences illustrated by each theme. The themes were revisited many times and reorganised until all researchers were satisfied that the data were represented in a coherent and valuable way [22]. The final themes and subthemes were defined and named. Lastly, the researchers wrote the data findings in a concise, coherent, logical, non-repetitive and interesting account of the experiences within and across themes [21]. As a result, a total of three themes were established. Subsequently, the results based on the established themes were written.

### Research Rigour

To ensure research rigour, trustworthiness and credibility methods were implemented. Investigator triangulation was done with two researchers conducting the focus groups to confirm results across researchers and decrease biases. All focus groups were transcribed manually and ratified by two other researchers to validate the data. An audit trail was implemented, including coding the raw data, generating themes and data analysis to confirm that the research results were recorded based on the responses and narration from participants and not from the researcher’s own ideas.

## Result

Three main themes were identified based on preclinical medical students’ experiences in utilising SC’s learning resources. The themes are *preparation for authentic clinical experience*, *accessibility of multiple resources for learning and support* and *opportunities to learn and improve*.

### Theme 1: Preparation for Authentic Clinical Experience

Establishing a realistic clinical experience for students in the SC prepares them for the actual clinical practice. The three subthemes emerged in preparation for authentic clinical experience are availability of physical and multimedia resources, realism of resources and patient-centred care. Students highlighted that availability of physical and multimedia resources in SC prepares them to deliver patient-centred care in a real setting. Examples of physical resources were mannequins and consumable items, and multimedia resources were videos that support students’ learning communication and clinical skills:

There are a lot of things, like catheterisation, DRE, you can’t find it, you can’t have that piece of machine or that mannequin anywhere else that I know. (FG1, 109–110)

They do have like videos and like manuals pasted near the venipuncture table, there is like a video, continuously being repeated and there is also a manual pasted. So, we can just check we are doing according to the steps. (FG1, 93–95)

Furthermore, students appreciated realistic resources in the form of SPs. They found communicating with SPs portraying an illness as useful as learning from the patients with real clinical signs, giving them an authentic experience. Nevertheless, students also highlighted their preferences for interacting with real patients:

For me it’s actually really good because some of the patients, some of the SPs, they really do act like they are sick. (FG3, 242–243)

And then they have like really enlarged thyroid right, we can really palpate and not just SPs like trying to feel. I feel like they should get real patients for taking […]. (FG2, 1124–1125)

During teaching sessions, students expressed that they were able to apply soft skills such as being empathetic and not being judgmental while communicating with the patients, which are instrumental in patient-centred care. This also reminds them to manage the patients as human beings and not as objects, thus improving patient care:

[…] I mean they act in the way that you really need to bring up a part of yourself to really like connect to them, especially like empathy stations. If they are good actors, then you feel like you care for them […]. (FG1, 249–251)

Basically, the idea was to explain our side of job is not to judge people, is their choices in life, but more like this is your patient, you are here to treat a human being. I think that’s what they are trying to get across. And also our opinion don’t matter when we talking to a patient. It is not about us, it is about them. I think that’s what we learn from that session. (FG2, 755–758)

### Theme 2: Accessibility of Multiple Resources for Learning and Support

The freedom to access resources such as space and equipment, as well as academic and peer support in the SC, enhances clinical and communication skills learning. The two subthemes emerged from the accessibility of multiple resources for learning and support, namely, psychological support and self-directed learning resources. Students perceived receiving support from their peers, peer tutors and academic staff of the SC as valuable resources for learning clinical and communication skills. They mentioned that their peers play a role by correcting their mistakes, helping fill in gaps in the learning process and are considered fun to study with. Students also find peers useful in practising their communication skills:

I think like one thing you can do with your friends, you both can, learn together and then your friends can teach you. Maybe sometimes you’ve missed like a step or two and then they will tell you all you should do this and this.. (FG3, 104–106)

Technically, we also learned communication by practicing procedures with our friend. we start to explain to them what to do with this the tool and how we are going to take your blood, we practice communication skills. (FG1, 171–173)

Other than peers, students find peer tutors who are their seniors as someone they can relate to since these tutors were recently medical students:

They were student before. They see it from the student point of view. So, I really enjoy the peer tutors. (FG1, 1010–1011)

I think that peer tutors understand us better because they’re like, not very much older than us compared to doctors, like the lecturers. So, the peer tutors are much more understanding of the fact that we don’t know everything yet like, you know, they were students just like us not very long ago. (FG3, 421–423)

Besides that, students shared that the academic staff at the SC also play a role in helping them practice their clinical skills. They turn to the staff for clarification and to clear doubts. Apart from this, students have also shared the need to standardise teaching across the academic staff:

But then we would also call the nurses and like double-check and see if what we are doing is correct or not. For example, […] during measuring blood pressure, I realised that there was one part we were doing it wrongly the whole time. And the nurses corrected us last minute before our test. (FG3, 121–124)

I think there needs to be a form of standardisation like for like teaching, because sometimes a lot of us get very confused […]. (FG3, 492–493)

The students find the resources in the SC useful for SDL. The availability of resources, freedom to practice and commitment to using the venue for self-practice are motivating factors that draw the students to the SC to brush up on their skills:

[…] there is no restriction to what you can or cannot do, you want to practice on mannequin hundred times, is up to you, can come in anytime to practice. (FG1, 74–76)

I think having the video in front really helps because sometimes we miss out one or two items, […]. (FG3, 76–77)

### Theme 3: Opportunities to Learn and Improve

The SC offers the privilege of experiencing repetitive practice and real-time feedback to improve students’ clinical and communication skills. The students agreed that hands-on practice in SC is essential for them to apprehend skills they learn during their small group teaching sessions. They feel that this provides an opportunity for all students in the group to take part in interviewing patients. Students also expressed that the self-practice session creates the occasion for them to learn from each other’s mistakes:

*Because I think when the lecturer calls you up, the student will have to do it. And then everybody can learn […]. (FG3, 392–393)*

*[…] So that we actually know like each other mistakes, we will take turns to be the SP and be the one who do the physical examination. (FG3, 145–146)*

The students deemed receiving real-time feedback a valuable element in their learning as it helps them improve their skills. This shows that students prefer to be corrected immediately during the teaching sessions by the facilitators:

[…] I will volunteer to do the procedure and do history taking. And the lecturer also give very good suggestions for me to improve in my skills. (FG3, 165–166)

Because in the end the nurses, they’ll give us feedback. And like they will tell us like how we did, how it goes. So, it’s a good learning process […]. (FG3, 487–488)

The students also value peer feedback as it helps each other learn through communication and sharing of knowledge:

[…] a few people like to practice together because we don’t want to waste time so we won’t practice in a big group, but we will like separate into small groups. Then after that we will just cross check with each other, see if each other is doing correctly […]. (FG3, 115–117)

## Findings of the Study in Relation to the Kolb’s Experiential Learning Cycle

**Fig. 2:**
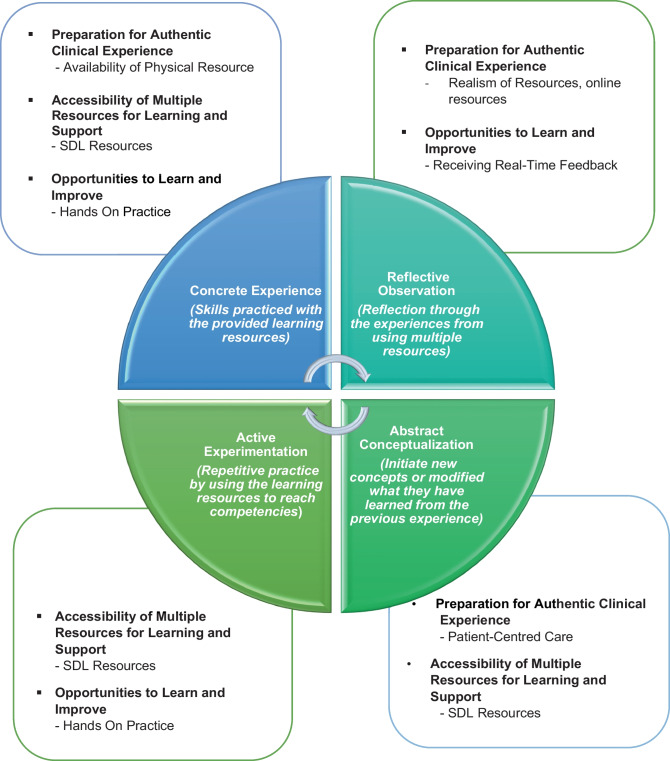
Findings of the study in relation to the Kolb’s experiential learning theory

Kolb’s experiential learning cycle was proposed as a theory to view the students’ experiences in utilising the learning resources in SC. By overlapping the view offered in this theory with the study findings, we were able to gain a deeper understanding of the preclinical medical students’ experiences in utilising SC learning resources to support preclinical communication and clinical skills training. Figure 2 shows how learning resources in a SC support preclinical medical students’ learning using Kolb’s experiential learning cycle. The three themes identified in the study are mapped to the 4 stages of the Kolb’s experiential learning cycle with examples of learning activities.

## Discussion

The three themes identified from this study and the lens of the Kolb’s experiential learning cycle have addressed the research question ‘How are the SC resources useful in supporting preclinical medical students' clinical and communication skills learning?’ We posit that educators who design SC activities should consider, contextualise and communicate the themes prior to implementation. The themes, preparation for authentic clinical experience, accessibility of multiple resources for learning and support followed by opportunities to learn and improve, are important as it shows the purpose, means and outcomes of simulation learning from the preclinical perspective. These themes are in support of previous findings related to the use of simulation centres in the medical curricula irrespective of type of undergraduate clinical programmes or institutional resource settings (5, 9, 12). The additional insight from this study is that preclinical medical students share similar views on how SC support clinical and communication skills learning and that there are beneficial outcomes of having SC sessions either facilitated or self-directed in year 1 and 2. The themes, the associated learning theories, resources used and practical benefits are discussed in the following sections.

## Preparation for Authentic Clinical Experience

The availability of physical and multimedia resources prepares students for authentic clinical experiences. According to Kolb’s experiential learning cycle, concrete experience to practice clinical and communication skills allows students to have hands-on experience with new skills or practice the same skills from different perspectives (Fig. 2). An example from this study would be video-assisted learning; students see it as a valuable tool to practice and improve skills. Video-assisted learning promotes self-directed learning as they use them to enhance their comprehension and cognitive ability. Examples of video-assisted learning in our settings are task trainers like venepuncture, electrocardiogram (ECG), injection pad, breast model, antenatal model and digital rectal examination (DRE) models combined with videos that show these procedures available for students to access in the self-directed learning area in the SC (https://www.imu.edu.my/self-directed-learning-sdl/). Studies have previously reported that readily available educational video resources are an added instructional tool for students to practice skills and enable repetitive skills to reach clinical competency [23, 24]. Our findings highlight the benefits of these resources for preclinical students through a self-directed learning approach.

Students also highlighted that the realism of resources was crucial in duplicating cases seen in real clinical settings. This means SCs should strive to provide diversity of scenarios with patients from diverse backgrounds, use of props and technologies that can add to the realism of a case during clinical sessions [25]. Similarly, encounters with SPs are worthwhile and give students realistic patient encounters. William and Song [26] reiterated that utilising SP in medical education enhances students’ performance. Furthermore, each SP role can be adapted to specific learning purposes, and structured SP training is widely recommended to provide consistent portrayal in teaching and assessment [27]. However, developing the program requires cost, workforce to plan, execute and quality assurance [28]. The SP program at our institution has been established for nearly 30 years. Internal evaluations suggest that investment in and rethinking SP training and roles is as important as curriculum review. Other than realism, students felt the SP help them empathise through a patient-centred approach. According to Wilcox et al. [29], patient-centred care can be taught through an explicit curriculum with a formal learning method. Patient-centred care is defined as treating the patient as a unique human being, including psychological and social aspects, and not only the disease [30]. Curriculum design includes various scenarios to create awareness and changes toward a non-judgemental attitude, acceptance, empathy and confidence to communicate with the community respectfully [31]. Additional insights from this study show that preclinical students appreciated the realism of the scenarios and the patient-centred resources at SC. This appreciation supports the reflective observation and abstract conceptualisation stage of the Kolb’s cycle (Fig. 2), highlighting further the importance of designing contextual healthcare-relevant scenarios that addresses socio-cultural issues early in the curriculum. To achieve this, it is proposed preclinical academic staff in simulation centres need to have access to and continue to serve as practitioners in healthcare settings.

## Accessibility of Multiple Resources for Learning and Support

The preclinical students identified accessibility is essential for learning and support. Accessibility as shown in other studies [9, 32] relates to opportunities to have hands-on experiences, practice time and opening times and the preclinical students in this context also highlight the importance of this. Studies on simulation with medical students do highlight the importance of accessibility to multiple resources including high-fidelity simulators, simulated patients and task trainers; however, accessibility to SC facilitators as resource is less mentioned [9, 11, 32].

A key finding of this study is that preclinical students highlighted peers, near peers and faculty including medical and nursing staff as an important resource of the CSSC. This concept warrants future studies as it provides the opportunity for a rethink of SC beyond expensive simulators or technological requirements with a refocus on the people that are part of it as important resources too. This is because psychological safety serves as a foundation for effective learning. In simulation-based education, it is important for students to be able to express themselves physically, cognitively and emotionally without the fear of negative consequences. However, simulation training can contribute to psychological distress for students in areas such as having their performance recorded, reviewed and graded in front of peers. Apart from that, past negative simulation experiences may also hinder the students’ learning processes and performance [32, 33].

The perception of peers or near peers as resources help students initiate self-directed learning as it leads to practising with a group of friends. The friends take turns to be SP, and they assist by correcting each other’s mistakes and providing feedback for improvement. This forms part of active experimentation in the Kolb’s cycle and is a reiterative process that enhances the next experience. Our findings are supported by a study on medical students’ perception of receiving peer feedback. Burgess and Mellis [34] stated that the students believed their peers had sufficient knowledge to provide useful feedback and found that the quality, honesty and correctness of the feedback improved when it was given outside of the classroom and in a more relaxed environment. In learning clinical skills, having a critical peer gives new dimensions for students to reflect. A critical friendship allows the ‘friends’ to recognise their flaws in clinical and pedagogical approaches based on friendship and mutual trust [35]. Based on our findings, it is essential to create an environment in the SC where students may freely study clinical skills with peers to support each other.

Similarly, peer tutors who are senior students are viewed as valuable resources to teach and guide junior students*.* Peer tutoring is described as ‘uses students as tutors who are not professional teachers helping each other learn and learn themselves by teaching’. Medical students felt more comfortable asking questions when taught by peer tutors. Both groups have a sense of belonging with common interest and objectives, promoting supportive interaction and fostering confidence for tutors and tutees alike [36]. Studies have shown that medical students preferred to learn clinical skills taught by peer tutors compared to faculty [37] and there were no significant differences in peer tutor compared to faculty teaching [38]. In IMU, a comprehensive peer tutor program that involves training, continuous evaluation and feedback was developed nearly a decade ago, and our findings suggest that this initiative needs to be continued and supported in SC.

The academic staff at a SC is also regarded as individuals who facilitate students in learning clinical skills. Besides teaching, our result supports other literature where students look up to them as role models, have clinical knowledge and personal attributes and value their supervision and guidance [39, 40]. However, during this study, the researchers also found that standardising teaching among academic staff is a challenge. The students have highlighted this during the focus group as they claim they are confused with the inconsistent delivery of teaching from different academicians. This finding is similar to the study by Lambert et al. [41], which emphasises standardisation and importance of consistency in teaching quality and approaches in medical education so that all students reach an agreed-on, expected level of proficiency in both knowledge and skills.

Students had a positive perception that the resources in IMU SC’s self-directed learning space helped prepare them for the actual clinical experience in the future. They feel that SDL gives them the freedom to plan their learning in terms of schedule, topics to practice and who they practice with. They like the fact that they can practice in a relaxed atmosphere and have access to the equipment required, a shortcoming identified in other studies too [9, 11, 32]. The SDL enables them to mingle with peers and improve clinical and communication skills in non-formal but safe environments.

## Opportunities to Learn and Improve

When equipped with all the necessary resources, the SC provides the opportunity for students to engage in practical revision and receive feedback for improvement. Two key concepts that are core to the Kolb’s experiential learning cycle appear in this theme (Fig. 2), *hands-on* practice and *real-time* feedback.

Hands-on practice is crucial for medical students to improve the skills learned during the teaching sessions. Khan et al. [42] stated in their study that hands-on practice is the principal part of medical students’ learning. According to them, it prepares them with the knowledge of basic clinical skills required to enter the healthcare environment. They also emphasised that the quality of their hands-on practice will determine the students’ dexterity to deliver quality care to the patients in the future. From our study, we also found that students obtain the opportunity to improve their skills at their own pace during hands-on practice. Roy et al. [43]. posited that students who do more hands-on practice become more professional, perform procedures faster, develop better communication skills and provide better medical care to the patients.

Real-time feedback is defined as any positive or constructive statements about clinical performance shared between trainees and supervisors shortly after an observed behaviour. Real-time feedback is formative and requires setting expectations, so the learner is prepared to receive feedback in this manner [6, 12, 44]. This study also highlights that apart from real-time feedback, preclinical students view frequent and multiple sources of feedback (peer, near peer and faculty) as a valuable element for learning in SC. Bugaj et al. [6] explained that the positive social and cognitive congruence between students and their peers or near peers helps in learning and receiving feedback as there is shared empathy due to similar social roles and ability to share knowledge and skills at a level that is understood better, cognitive congruence. Importantly as highlighted in previous studies [11, 12, 32], effective feedback is a resource outcome that can motivate students especially those in preclinical years to engage more in SC: *preparation for authentic clinical experience*, *accessibility of multiple resources for learning and support* and *opportunities to learn and improve*.

## Conclusion

In conclusion, the SC’s resources play a significant role for preclinical medical students, preparing them for authentic clinical experiences with a patient-centred care approach. Accessibility to multiple resources for learning and support in the SC created self-directed learning opportunities and enhanced social support from peers, peer tutors and academics. The resources allowed for opportunities to learn and improve, evidence that the preclinical students learning is aligned to the 4 stages of the experiential learning cycle. Importantly, the study highlights the importance of a safe learning environment to aid the achievement of competencies including clinical knowledge, skills and attitudes. Therefore, SC must continue to enhance its resources to keep up with current trends in healthcare and meet students’ learning outcomes.

## Limitations

There are several limitations to this study. Focus group for the first and second groups was conducted before the pandemic at the end of 2019 and February 2020, while the third focus group was conducted at the end of 2020. Hence, we acknowledge that some participants may not have equal opportunity to access and experience the SC resources as others did. Nevertheless, all students in the study had access to SC resources.
